# Progress in Fevers

**Published:** 1901-09-21

**Authors:** 


### PROGRESS IN FEVERS.

(*Continued from page* 399.)

**Plague.**—Alex. Edington[37] records his experiments made under Government in Cape Colony regarding the identity of rat plague and bubonic plague. A careful post-mortem examination was made of a rat found recently dead in the dock. Naked eye examination revealed merely a little fluid in the pleura and pericardium, congestion of the liver, and pallor and enlargement of the adrenals. No enlarged glands were found. Microscopical examination of the blood showed enormous numbers of bacteria present also in the spleen and liver juice. These bacteria were oval and frequently joined as diplo-bacteria. Preparations from the spleen stained with methyl-violet were compared with similar preparations of the pus taken from the buboes of plague patients in Capetown. The plague organisms showed well-marked bipolar staining not observable in those of the rat. Cultures of the rat organisms on agar agar were more profuse than those of plague bacilli. The primary culture of the former showed diplo-cocci and diplo-bacteria and large irregular spherical cells. On further culture the rat and plague organisms were indistinguishable. The latter, but not the former, gave an acid reaction when inoculated on litmus agar. Inoculation of guinea-pigs with rat organisms caused death, accompanied by œdema, and enlarged glands and spleen. The spleen was covered with the small white points seen in guinea-pigs killed by inoculation with bubonic plague. Bacteria were abundantly present. A baboon and two rabbits inoculated similarly with rat bacteria gave no sort of reaction, but the two rabbits died after subsequent inoculation with bubonic plague. Post-mortem œdema, enlarged glands and spleen, and congested liver covered with white points were found. The liver and spleen yielded pure cultures of the bacillus. A guinea-pig inoculated at the same time showed similar changes. Rabbits inoculated with a culture of rat bacteria passed through a guinea-pig showed no characteristic signs when killed, nor were bacteria found in the blood or organs. Pigeons inoculated with a culture of rat bacteria died without very characteristic signs, but pure cultures of the bacteria showing well-marked bipolar staining were obtained from the blood and liver. From a consideration of these facts "it seems clearly proved that this rat plague cannot be bubonic plague." Regarding the infectivity of plague, Klein[38] points out that it depends on the type of the disease. In the pneumonic form the expectoration teems with bacilli; in the septicæmic, or hæmorrhagic, type they swarm in the blood; in the bubonic variety they are practically limited to the spleen and lymphatic glands, and until pus escapes from the glands the chance of infection is slight. This explains why the disease was not spread by the seaman who journeyed by rail from the Tyne to Landaff. Klein finds that guinea-pigs are not affected by inoculation with dead cultures of plague bacilli. If the blood of this animal is prepared by injecting small doses of living cultures it shows an agglutinating action on salt emulsions of plague bacilli obtained from patients in all parts of the world, and also on plague cultures obtained from rats. Normal blood—human or animal—does not give this reaction.

**Malaria.**—Prof. Celli[39] finds that the larvæ of anopheles will not live in salt sea-water, nor in very brackish or stinking putrid water. Running water is also prejudicial to their growth, hence drainage canals should be flushed every fortnight. Manson[40] advises the study of mosquitoes in non-affected as well as in infected places, with a view to determining the causes of the seemingly capricious distribution of malarial varieties. Nuttall[41] offers a similar suggestion, and points out that the assumption of Grassi that the geographical distribution of anopheles coincides with that of malaria does not hold good for England, where anopheles is found in non-malarial districts, and also in districts formerly malarial. Wright[42] finds that the larvæ of mosquitoes in Aberdeenshire are extremely resistant to severe cold. Lo Monaco and Panichi[43] find that quinine seems to act in strong solution by killing the parasite whilst it is attached to the red corpuscle, in weaker solution by setting it free into the blood plasma to be devoured by phagocytes. Iwanoff[44] finds that methylene blue attacks the protoplasm especially, while quinine has a special affinity for the nucleus and chromatin of the malarial parasite. Hence methylene blue has a rapid destructive action on the adult parasites and on the crescents, which are chiefly composed of protoplasm, and which are consequently very refractory to quinine. Bluemchen[45] finds that quinine can be injected subcutaneously without resulting local lesions by dissolving the muriate in hot water in the syringe and injecting at a temperature of 98° Fahr. A hot compress over the site of injection facilitates absorption. Crespin and Mailfert[46] find that acute bronchitis, especially at the left base, is very frequent in malaria. The bronchial complications seem proportional to the splenic and hepatic lesions. Pulmonary congestion is common and this may predispose to pnuemonia, which, although it yields best to quinine, is of the pneumococcus variety. An apical consolidation simulating phthisis is often present.

[37] Lancet, June 8. [38] Lancet, June 1. p. 1541. [39] Amer. Jour. Med. Sc., May. [40] Ibid. [41] Med. Rec., May 4. [42] Brit. Med. Jour., April 13. [43] Brit. Med. Jour., April 20. [44] Med. Rec., May 25. [45] Ibid. [46] Brit. Med. Jour., May 18.

